# Stereotactic Body Radiation Therapy for Biopsy-Proven Primary Non–Small-Cell Lung Cancer: Experience of Patients With Inoperable Cancer at a Single Brazilian Institution

**DOI:** 10.1200/JGO.18.00020

**Published:** 2018-07-26

**Authors:** Carlos E.C.V. Abreu, Fabio Y. Moraes, Fabiana A. Miranda, Gabriela S.M. Siqueira, Rafael Gadia, Cecilia K. Haddad, Heloisa A. Carvalho

**Affiliations:** **Carlos E.C.V. Abreu**, **Fabio Y. Moraes**, **Fabiana A. Miranda**, **Gabriela S.M. Siqueira**, **Rafael Gadia**, **Cecilia K. Haddad**, and **Heloisa A. Carvalho**, Hospital Sírio–Libanês; **Heloisa A. Carvalho**, Universidade de São Paulo, São Paulo, Brazil; **Fabio Y. Moraes**, University of Toronto, and Princess Margaret Cancer Center, University Health Network, Toronto, Ontario, Canada.

## Abstract

**Purpose:**

Stereotactic body radiation therapy (SBRT) has emerged as a treatment option for patients with non–small-cell lung cancer (NSCLC). We report the clinical outcomes and toxicity for patients with inoperable primary NSCLC treated with SBRT.

**Methods:**

Between 2007 and 2015, 102 consecutive lung lesions were treated with SBRT at our center, of which 59 primary NSCLC lesions (from 54 patients with inoperable disease) were retrospectively reviewed (43 lesions were excluded because of metastases or because there was no biopsy specimen). We report infield local control (LC) per SBRT target, regional or distant failure-free survival, and overall survival (OS) per patient, using Kaplan-Meier estimates. Serious toxicity was retrospectively scored using Common Terminology Criteria for Adverse Events, version 4.

**Results:**

Most of the 54 patients were men (n = 41; 76%), median age was 75 years; stage IA (n = 36; 66%) and adenocarcinoma (n = 43; 80%) were the most common stage and histologic diagnosis, respectively. Five patients had two lung lesions. A median of three fractions (range, 3 to 5 fractions) and a total median dose of 54 Gy (range, 45 to 60 Gy) per lesion were prescribed. The median follow-up was 17.8 months (range, 4 to 56.4 months). The 2-year rates of LC, regional or distant failure-free survival, and OS were 89.1% (95% CI, 72.2% to 96%), 79% (95% CI, 59.8% to 89.8%), and 80% (95% CI, 64% to 89.8%), respectively. Grade 3 to 4 toxicities were observed in two patients (3%): grade 3 pneumonitis (n = 1) and grade 4 skin toxicity (n = 1).

**Conclusion:**

SBRT results in high rates of 2-year LC, regional or distant failure-free survival, and OS with low rates of severe toxicity in patients with inoperable primary NSCLC disease.

## INTRODUCTION

Lung cancers are common and are associated with high death rates in developed and nondeveloped countries.^1^ Most patients with lung cancer will be diagnosed with non–small-cell lung cancer (NSCLC) and 15% to 20% will present with stage I disease.^2,3^ Historically, the standard treatment of patients with stage I NSCLC is lobectomy or pneumonectomy, with a 5-year overall survival (OS) rate of 60% to 70%.^2^ Radiotherapy (RT) and chemotherapy were considered adjuvant or palliative treatments. However, in the past 10 years, the rapid development of RT and imaging technologies has allowed increased safety and efficacy of the use of stereotactic body radiation therapy (SBRT) for more indications, including lung cancers.^4-6^

SBRT is a noninvasive method used to deliver a high ablative dose of ionizing radiation to a small tumor volume with a few fractions (generally no more than 8 fractions) under image guidance and using methods of controlling internal tumor movement.^4,7,8^ Institutional series of SBRT report high local control (LC) rates, reaching 95% in small (≤ 5 cm in the largest diameter) peripheral tumors and negative nodes.^4,5,9-12^ Initially there was concern regarding the use of SBRT in the treatment of central lung lesions (defined as a lesion within 2 cm of the bronchial tree).^13^ A systematic review of 563 central lung lesions treated with SBRT^14^ reported grade III or IV toxicity rates < 10% and a treatment-related mortality rate < 5%.

Thus, we aimed to evaluate and report a single Brazilian institution’s experience in the use of SBRT for the treatment of patients with medically inoperable, biopsy-proven, primary NSCLC, because there are limited reports of this approach outside of developed countries.

## METHODS

This was a retrospective study, approved by the institutional review board and carried out in the Radiation Oncology Department of the Sírio-Libanês Hospital (São Paulo, Brazil). The study population consisted of consecutive patients who presented with biopsy-proven NSCLC, early stage (ie, T1 to T2 N0M0), T3N0M0 (ie, more than one lesion in the same lobe) or T4N0M0 (ie, more than one lesion involving distinct lobes in the ipsilateral lung), according to the Union for International Cancer Control TNM Cancer Staging Manual, 7th edition.^15^ Metachronous tumors confirmed by biopsy specimen evaluation were also included. All patients were considered inoperable by a multidisciplinary team or declined surgery.

### SBRT

For the administration of SBRT, an in-house semirigid device for positioning and immobilization of the patients was developed. Later (from June 2012), commercial devices were used (BodyFIX; Elekta, Stockholm, Sweden). The immobilization device was indexed to the patient’s body and treatment couch. We performed internal organ and tumor movement analysis with three consecutive computed tomography (CT) sequences: normal breathing, forced inspiration, and forced expiration and/or CT with slow image acquisition. In April 2014, four-dimensional CT (4DCT) imaging was applied. With this approach, we defined the internal target volume by personalized assessment of tumor motion. A standard margin of 0.5 cm to 1.0 cm was added to the internal target volume to create the planning target volume. Planning target volume margins were 0.5 cm in all directions, except inferior and superior before 4DCT implementation, and 0.5 cm in all directions for all patients treated with 4DCT.

The pretreatment positioning of the tumor and patient was evaluated with cone beam CT images. Tumor and patient displacements were corrected immediately before each SBRT fraction by cone beam CT. All patients were treated with three-dimensional conformal RT, noncoplanar beams, and stereotactic technique. The treatment planning followed the protocols of the Radiation Therapy Oncology Group clinical trials 0236 (ClinicalTrials.gov identifier: NCT00087438) or 0813 (ClinicalTrials.gov identifier: NCT00750269). A 2-cm perimeter around the proximal bronchial trees, per Radiation Therapy Oncology Group guidelines,^16^ wasused to define central and peripheral lesion location. Dose and fractionation were defined considering tumor location, size, and current available evidence: three fractions for peripheral lesions and three to five for central lesions.

### Clinical Outcomes

The primary outcome was OS. Secondary outcomes were local failure-free survival (LFFS), regional or distant failure-free survival , and toxicity profile. All outcomes were assessed from the date of delivery of the first SBRT fraction to last follow-up or death. Local failure or recurrence was defined in the presence of one of the following criteria: (1) CT imaging with increasing consolidation over time mass size without inflammatory signs; (2) positron emission tomography/CT study with increased standard uptake value greater than expected for lung injury (ie, ≥ 5); and/or (3) biopsy specimen positive for a lesion. Acute (≤ 6 months) and late (> 6 months) toxicity rates were assessed and defined based on the Common Toxicity Criteria for Adverse Effects, version 4.0.^17^

### Statistical Analysis

Descriptive analysis of patients and lesions was performed. The qualitative variables were summarized by frequency and percentage, and the quantitative variables by mean, standard deviation, median, minimum, maximum, and number of valid observations. Estimates of survival probability were calculated by the Kaplan-Meier method. Statistical significance was set at *P* < .05. Statistical analysis was performed using Stata, version 13.0 (StataCorp, College Station, TX).

## RESULTS

### Sample Characteristics

Between January 2007 and September 2015, 102 lung lesions were consecutively treated with SBRT in our institution. Forty-three lesions were excluded for being > 5 cm, metastatic, or not biopsy-proven NSCLC. Patients with a diagnosis of idiopathic pulmonary fibrosis or under treatment for other cancer at the time of SBRT assessment were not included. The final sample comprised 59 NSCLC lung lesions (n = 54). Patients’ characteristics and cause of inoperability are listed in Tables 1 and 2, respectively.

**Table 1:**
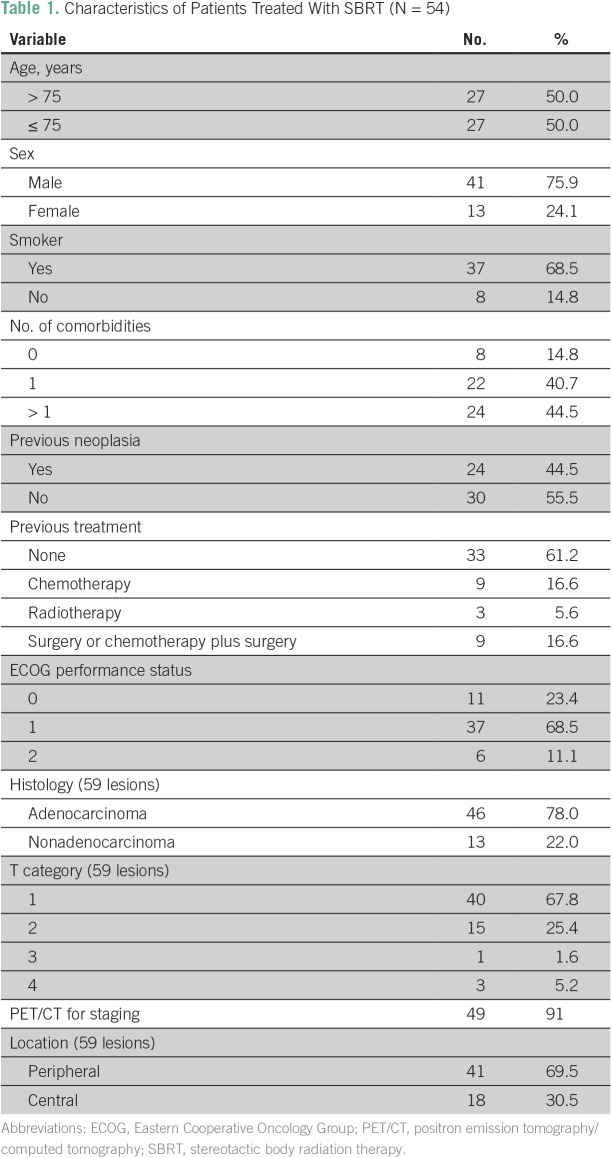
Characteristics of Patients Treated With SBRT (N = 54)

**Table 2:**
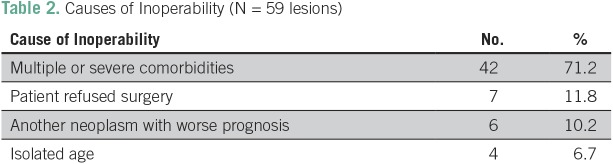
Causes of Inoperability (N = 59 lesions)

The age range of the cohort was 55 to 96 years (median, 75 years). The median Eastern Cooperative Oncology Group performance status was 1. The most common SBRT dose schema was three fractions at 18 Gy, which was administered to 29 (49%) of the 59 lesions followed by three fractions at 15 Gy, which was administered to 16 (28%), four to five fractions at 10 Gy, which was administered to eight (21%), and three fractions at 20 Gy, which was administered to six (15%). The median biologically effective dose (BED)_Gy10_ of the entire cohort was 112 (range, 80 to 180) and only two patients (3.3%) had a BED_Gy10_ < 100. Lesions were generally considered inoperable because of patients’ multiple comorbidities. A total of 24 patients had history of previous cancer (Table 3).

**Table 3:**
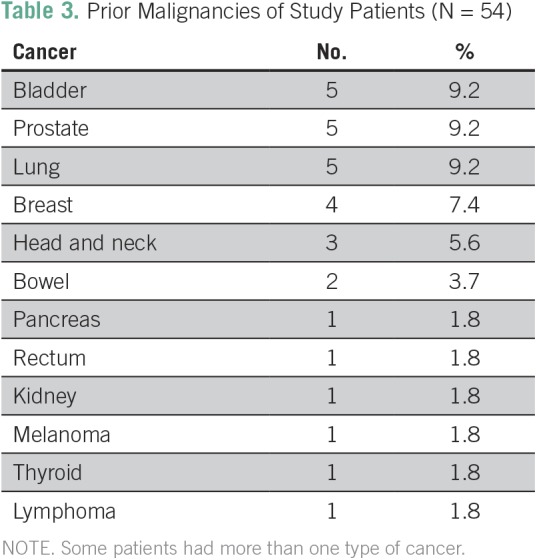
Prior Malignancies of Study Patients (N = 54)

### OS

**Fig 1:**
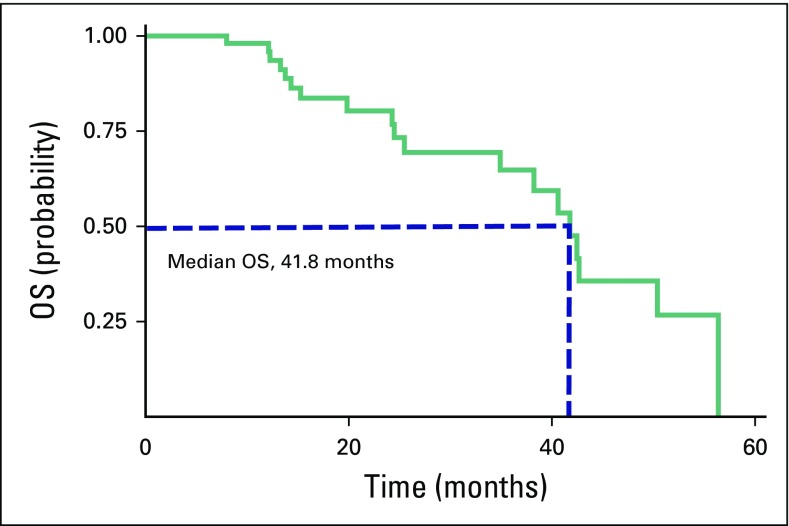
Overall survival (OS) Kaplan-Meier estimates (n = 54 patients).

Patient follow-up ranged from 4.2 to 56.4 months (median, 18.7 months). Nineteen patients (35%) died during the follow-up period, four specifically of lung cancer and 15 of causes not related to lung cancer. The median OS was 41.8 months (95% CI, 39.4 to 50.4 months; Fig 1). Eight patients (15%) died within the first 24 months after the SBRT; the 2-years OS was 80% (95% CI, 64% to 90%). Twelve patients (22%) died within 36 months after SBRT; 3-year OS was 64.8% (95% CI, 45% to 79%).

### LFFS

**Fig 2:**
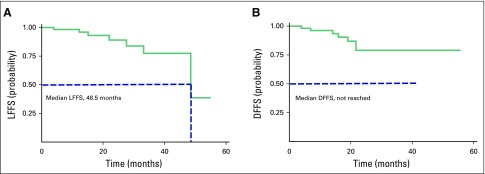
Kaplan-Meier estimates. (A) Local failure-free survival (LFFS) (n = 59 lesions); (B) Regional ordistant failure-free survival (DFFS) estimates, (N = 54).

For LFFS evaluation, 59 lesions from 54 patients were considered. The lesion follow-up time ranged from 3.9 to 55.0 months (median, 16.8 months). Local treatment failure over the follow-up period was seen in seven lesions (12%); the median time to LFFS was 48.5 months (Fig 2A). Four lesions (7%) showed local failure within 24 months after SBRT; the 2-year LFFS was 89% (95% CI, 72% to 96%). Local treatment failure occurred in six lesions (10%) within 36 months after SBRT; 3-year LFFS was 77% (95% CI, 53% to 90%). Only one patient underwent a biopsy of a locally recurring lesion and received radiofrequency ablation as salvage treatment.

### Regional or Distant Failure-Free Survival

Patient follow-up time for regional or distant failure-free survival ranged from 3.9 to 55.8 months (median, 17.6 months). During the follow-up period, seven patients (13.0%) had regional or distant failure (regional failure (n = 2), distant failure (n = 3), and both regional and distant failure (n = 2). The median time was not reached (Fig 2B). All regional or distant failures occurred within 24 months after SBRT, and 2-year DFFS was 79.0% (95% CI, 59.8% to 89.8%). Distant failure occurred in the following locations: liver and bone, bone and brain, liver and peritoneum, and, in two patients, bone alone. For isolated regional failures, no regional salvage treatment was performed.

### Toxicity

An acute toxicity event occurred in 21 (39%) of the 54 evaluated patients and a late toxicity event occurred in eight (15%) of 54 patients. Table 4 summarizes data on acute and late toxicity events. Acute or late events of grade > 2 were reported in two (3.7%) of the 54 patients (grade 3 pneumonitis [n = 1] and grade 4 radiation dermatitis [n = 1]). We believe the latter could represent a mix of decubitus ulcers and radiodermitis.

**Table 4:**
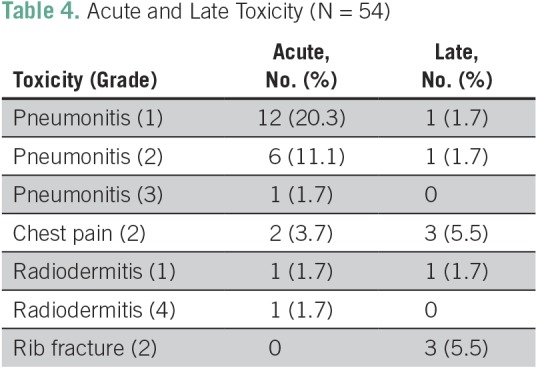
Acute and Late Toxicity (N = 54)

## DISCUSSION

Our results highlight that the use of SBRT for NSCLC treatment in patients with inoperable lesions is safe and provides prolonged median survival (41.8 months), high 2-year OS (80%), and good LFFS rates (89% at 2 years) in a non-North American or European institution. SBRT and protracted RT (hypofractionation) are technical and biologic advances with the potential to help close the RT gap between the need for and access to RT.

Regarding external validity, our results are comparable to those of important, selected, prospective series that included patients with pathologic confirmation of cancer (Table 5). Previous studies, also demonstrated that LC is strongly associated with BED_Gy10_ > 100.^18,19^ We reported an LFFS of 89% with median BED_Gy10_ of 112 Gy and DFFS of 79%. According to a recent review of stage I NSCLC treated with SBRT, regional recurrence rates were between 4% and 17%, and distance recurrence rates were between 8% and 34% in the first 3 years, which are consistent with our results.^20^

**Table 5:**
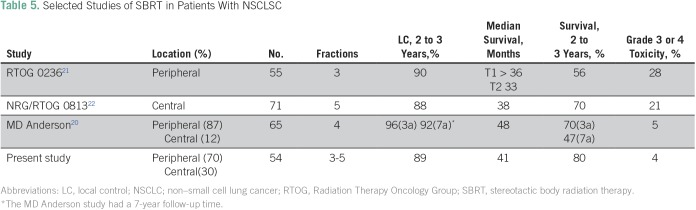
Selected Studies of SBRT in Patients With NSCLSC

Differences in OS between our study (median, 41.8 months) and others are possibly related to patient selection. It is important to highlight that the patients in our study had a poor prognosis; half of the cohort was older than 75 years of age, four patients had stage T3 or T4 disease, only 12% were fit patients who refused surgery, and 44% had previous cancer (Tables 1 and 3). In addition, a retrospective study with data from a tumor registry involving 3,147 patients showed a median survival of 10 months in patients with untreated, early-stage NSCLC and 29 months in patients who received SBRT. That study shows that this gain persists independently of age, even in patients > 85 years of age, which encourages us to keep using SBRT treatment on our population.^23^

Half of our patients experienced some adverse effect, predominantly treatment-related pneumonitis (38%). Transient chest pain was the second most frequent adverse event requiring medication (9%). There were few cases of rib fracture (5%) and one case of severe radiation dermatitis, but no fatal event. The low rate of serious adverse events, especially in a sample that included 30% central lesions, is possibly related to the absence of patients with pulmonary fibrosis and few patients who received prior thoracic RT (n = 3), all conditions known to correlate with higher rates of toxicity.^24,25^ The absence of 4DCT for RT simulation in 70% of the patients in our series could be of concern. Nevertheless, we consider this the highlight of our study, demonstrating that even in this setting, SBRT can be performed with appropriate volume definition and planning, with the availability of an image-guided RT system for patient setup.

The main limitations of our study are related to the retrospective design, limited sample size, several SBRT dose schemes, and low statistical power for comparison between groups. In addition, toxicity rates should be interpreted with caution because of the retrospective analysis, which could lead to bias or underestimation.

Considering the current literature, lung SBRT is a highly effective modality, with survival rates up to three times higher when compared with observation,^14^ for treatment of early-stage NSCLC in patients with poor performance status. This dramatic improvement in clinical outcomes is uncommon in oncology, even more so in a group with unfavorable factors.

In a single Brazilian institution, the use of SBRT in patients with inoperable early-stage NSCLC demonstrated high levels of LC and OS with a favorable morbidity profile in patientswho had unfavorable factors for disease treatment. These data support the continued use of this technique in our clinical practice and could be an incentive for other institutions in developing countries.
